# Association of Cigarette Smoking History With Drug Abuse History and Arterial Carboxyhemoglobin in Trauma Activation Patients: A Retrospective Study

**DOI:** 10.7759/cureus.58606

**Published:** 2024-04-19

**Authors:** C. Michael Dunham, Gregory S Huang, Elisha A Chance, Barbara M Hileman

**Affiliations:** 1 Trauma, Critical Care, and General Surgery, St. Elizabeth Youngstown Hospital, Youngstown, USA; 2 Trauma and Neuroscience Research, St. Elizabeth Youngstown Hospital, Youngstown, USA

**Keywords:** trauma, drug abuse, carboxyhemoglobin, cigarette smoking, trauma centers

## Abstract

Introduction

The relationship between cigarette smoking and arterial carboxyhemoglobin (CoHb) in trauma activation patients has not been investigated. The aim was to determine if cigarette smoking is associated with drug abuse history and arterial CoHb levels.

Methodology

This is a retrospective review of level I trauma center activations aged 18-60 during 2018-2020. A medical record audit was performed to assess each patient’s cigarette smoking and drug abuse history and admission arterial CoHb level. The CoHb levels and smoking history for each patient were used to construct a receiver operating characteristic curve.

Results

Of the 742 trauma activations aged 18-60, 737 (99.3%) had a documented cigarette smoking history. Smoking history was positive in 49.7% (366) and negative in 50.3% (371). The positive smoking proportion was greater in patients with a drug abuse history (63.9% (234/366)) than those with a negative history (31.0% (115/371); p<0.0001; odds ratio=4.0). In 717 patients with a CoHb value, the CoHb was higher in smokers (3.9±2.2%) than in non-smokers (0.5±0.4%; p<0.0001; Cohen d=2.2). A CoHb >1.5% was higher in smokers (93.3% (333/357)) than non-smokers (1.7% (6/360); p<0.0001; odds ratio=818.6). The receiver operating characteristic curve for the relationship between CoHb and cigarette smoking history showed an area under the curve of 0.980 (p<0.0001). Using an arterial CoHb level >1.5% to predict a positive smoking history and a CoHb level ≤1.5% to predict a non-smoking history, sensitivity was 93.3% (333/357), specificity was 98.3% (354/360), and accuracy was 95.8% (687/717).

Conclusion

Cigarette smoking in trauma activations aged 18-60 is associated with drug abuse history and increased arterial CoHb levels on trauma center arrival.

## Introduction

The cigarette smoking history proportion in trauma center admission patients has been reported to be 48-53% in those with increased injury severity [1,2]. A positive smoking history in trauma center activation patients aged 18-60 has been shown to be associated with a positive blood alcohol content result, alcohol misuse, and a positive toxicology result [3,4]. Boehm, Cohen, Klesges, and Afsin have shown that carboxyhemoglobin (CoHb) levels are considerably higher in non-trauma patient cigarette smokers than in non-smokers [5-8]. One trauma patient study showed that an increased venous CoHb level was associated with a positive cigarette smoking history [9]. The major limitation of this investigation was that the trauma patients had “subcritical injuries” (the mean injury severity score was one) and were seen in the emergency department, but were excluded from the study if they required hospitalization [10]. A relationship between cigarette smoking and arterial CoHb in trauma activation patients has not been investigated.

An investigation of non-trauma participants showed that cigarette smoking is associated with a drug abuse history [11]. Cigarette smoking has been associated with an illicit drug use history in patients with minor trauma [9]. Because trauma activation patients at the current study institution routinely undergo arterial blood gas analysis upon trauma center arrival, we aimed to assess the association of cigarette smoking history with arterial CoHb levels. We also sought to determine if cigarette smoking and drug abuse history have a statistical association in trauma activation patients.

## Materials and methods

Study design and population

The parent group data source was adult patients admitted to Mercy Health Youngstown, a level I trauma center, during 2018-2020 as described in a previous publication [12]. In the current investigation, a consecutive subset of trauma activation patients (trauma team or alert status for patients with mid- or high-level injury acuity) aged 18-60 were included. Trauma activation patients aged 18-60 were selected because it has been found that this same group had an increased blood alcohol and toxicology positive proportion [3,4]. The medical record audit in the current study included an assessment of each patient’s cigarette smoking and drug abuse history and available admission arterial CoHb level. The method for classifying trauma activation patients aged 18-60 cigarette smoking history and drug abuse history (yes or no) have been described in detail in an earlier manuscript [4].

In particular, cigarette smoking history was obtained by performing an electronic medical record search for evidence of smoking. The terms smoking and smoker were separately entered into an electronic medical record search engine to detect relevant information. The statement was linked to the original documentation source in the electronic medical record and reviewed. Smoking history was typically recorded as never smoked, past smoker, or current smoking history. A positive smoking history was indicated as the current use of ≥0.5 packs per day. When electronic medical record documentation was sufficient, smoking history was categorized as yes or no.

A routine process was applied to determine the presence or absence of a drug abuse history. Drug abuse and substance abuse were separately entered into an electronic medical record search engine to detect relevant information. When a drug abuse history was documented to be present in the consultation or discharge summary records during the trauma center stay, drug abuse history was recorded to be positive. Further, toxicology screen results were routinely assessed for the 12 months before and after the trauma center stay. When an illicit drug was positive on the toxicology screen results, the drug abuse history was recorded to be positive. When drug abuse history was documented to be present or the toxicology screen was positive, the drug abuse history was categorized as yes. Otherwise, the drug abuse history was considered negative.

Arterial blood gas analysis at the time of trauma center arrival is a routine procedure for trauma activation patients at our institution. When available, the arterial blood gas CoHb value was collected from the electronic medical record. The CoHb levels and smoking history (yes or no) for each patient were used to construct a receiver operating characteristic curve. Statistical output from the software program analysis was used to identify dichotomous CoHb cut-points that would best discriminate smokers from non-smokers.

Statistical analysis

Continuous data (e.g., CoHb) are presented as the mean ± standard deviation and median, whereas categorical variables are reported as frequency count and percentage. Cohen d values were computed to assess the magnitude of two intergroup mean differences. For univariate analyses with a dichotomous dependent outcome (yes or no), intergroup mean differences were analyzed using the independent t-test for continuous data (e.g., CoHb). CoHb median differences for smokers and non-smokers were analyzed using the Wilcoxon rank sum test with a median test option. For dichotomous proportional data displayed in a 2×2 contingency table format (e.g., smoking history (yes or no)), a two-tailed Fisher exact test was employed to assess the odds ratio. The results were entered into Excel 2010 (Microsoft Corp., Redmond, WA, USA) and imported into SAS System for Windows, release 9.2 (SAS Institute Inc., Cary, NC, USA). For receiver operating characteristic curve analyses, data were exported from SAS into MedCalc® Statistical Software, version 19.2.6 (MedCalc Software Ltd, Ostend, Belgium). Significance levels for the p-value were set at <0.05.

## Results

The parent group consisted of 2076 consecutive trauma center admissions studied in the years 2018-2020 [12]. The 742 (35.7%) trauma activation patients aged 18-60 years were included in the current study. Of the 742 patients, 737 (99.3%) had sufficient information to classify patients with a positive cigarette smoking history (49.7%; n=366) or with a negative cigarette smoking history (50.3%; n=371). The positive cigarette smoking proportion was greater in male patients, patients sustaining penetrating trauma, and patients with a history of drug abuse (Table 1).

**Table 1:** Comparisons of cigarette smoking proportions by sex, mechanism of injury, and drug abuse history OR, odds ratio

	Smoking History	p-value	OR
Male	52.3% (281/537)	-	-
Female	42.5% (85/200)	0.0177	1.5
Penetrating trauma	61.9% (83/134)	-	-
Blunt trauma	46.9% (283/603	0.0017	1.8
Drug abuse history	63.9% (234/366)	-	-
No drug abuse history	31.0% (115/371)	<0.0001	4.0

**Figure 1:**
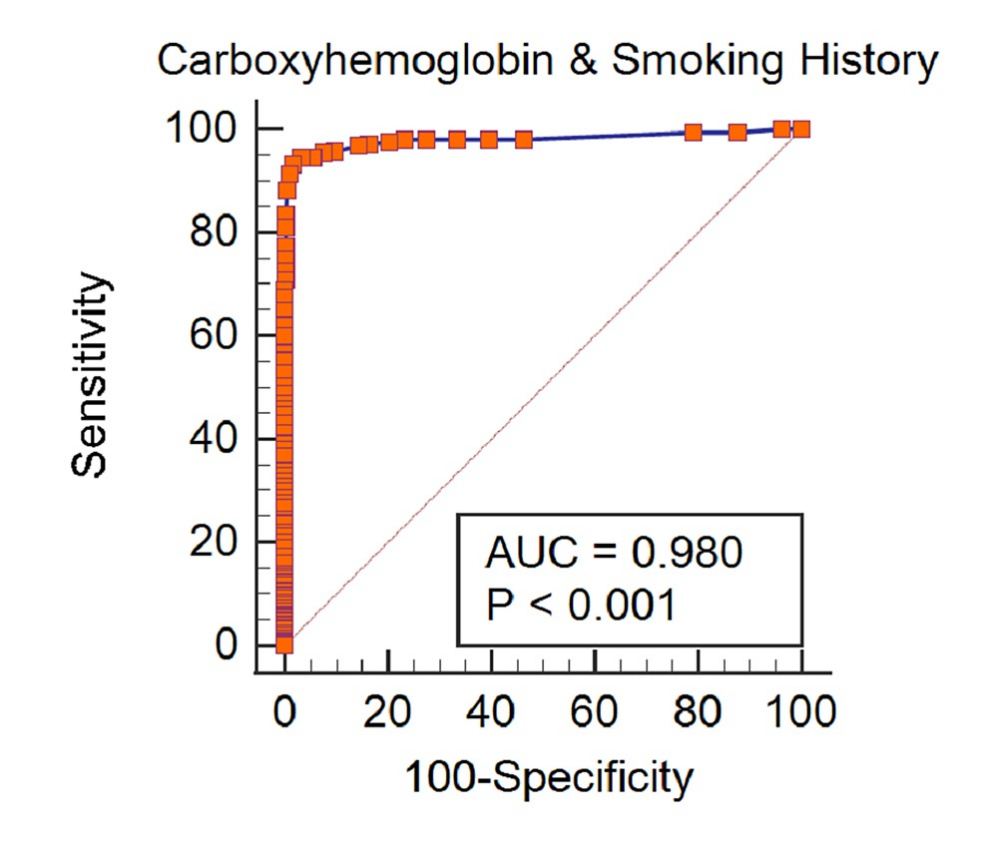
Relationship of arterial CoHb and cigarette smoking history AUC, the area under the curve; CoHb, carboxyhemoglobin

Of the 742 patients, 717 (96.6%) had sufficient information to classify patients with an admission arterial CoHb >1.5% (47.3%; n=339) or with an admission arterial CoHb ≤1.5% (52.7%; n=378). Multiple quantitative relationships demonstrated that patients with a positive cigarette smoking history had substantially higher arterial CoHb levels than those without a history (Table 2). The receiver operating characteristic curve for the relationship between CoHb and cigarette smoking history had an area under the curve of 0.980 (p<0.001; 95% confidence interval: 0.967-0.989) (Figure 1). The curve analysis indicated that a CoHb level >1.5% was most useful to identify patients with a smoking history, and a CoHb level ≤1.5% was best to detect patients with a non-smoking history.

**Table 2:** Comparisons of admission arterial CoHb levels between cigarette smokers and non-smokers OR, odds ratio; CoHb, carboxyhemoglobin

	Non-Smoker	Smoker	p-value	Cohen d	OR
Number	360 (50.2%)	357 (49.8%)	-	-	-
CoHb mean, %	0.5±0.4	3.9±2.2	<0.0001	2.2	-
CoHb median, %	0.3	3.4	<0.0001	-	-
Range	0-2.4	0.1-12.6	-	-	-
CoHb level >1.5%	6 (1.7%)	333 (93.3%)	<0.0001	-	818.6
CoHb level ≥2.0%	1 (0.3%)	289 (81.0%)	<0.0001	-	1525

The classification relationship between smoking history and CoHb is depicted in the 2x2 contingency shown in Table 3. When using a CoHb level >1.5% to predict a positive cigarette smoking history and a CoHb level ≤1.5% to predict a non-smoking history, sensitivity was 93.3% (333/357), specificity was 98.3% (354/360), and accuracy was 95.8% (687/717). The positive predictive value for using a CoHb level >1.5% to predict a positive cigarette smoking history was 98.2% (333/339). The negative predictive value for using a CoHb level ≤1.5% to predict a negative cigarette smoking history was 93.7% (354/378).

**Table 3:** Classification relationship outcomes for cigarette smoking history and arterial CoHb CoHb, carboxyhemoglobin

	CoHb >1.5% to Predict Smoker	CoHb ≤1.5% to Predict Non-Smoker
Smoking history, n	333	24
No smoking history, n	6	354

## Discussion

The positive cigarette smoking history in the current study of trauma activation patients aged 18-60 years was one in two, a finding similar to other investigations that studied trauma patients with increased injury severity [1,2]. A trauma activation study, which used serum cotinine as a surrogate marker for tobacco smoke exposure, found that tobacco use also occurred in a proportion of patients similar to that of the current study cohort with a positive cigarette smoking history [13].

The current study showed that the positive cigarette smoking proportion was modestly greater in male patients than in female patients. An increase in the proportion of male smokers has also been demonstrated in another trauma patient investigation [9]. However, both cohort investigations demonstrated that 40% of female patients have a positive cigarette smoking history. An investigation of a non-trauma cohort has also shown that smoking is more common in males than in females [14]. The current study demonstrated that the positive cigarette smoking history proportion was greater with penetrating trauma than with blunt trauma. Using serum cotinine as a surrogate marker for tobacco smoke exposure, other investigators have also shown that tobacco use is increased in patients with penetrating trauma [13]. The current study showed that positive cigarette smoking was greater in patients with a drug abuse history than in those with a negative history. A similar association has been described in a cohort of United States epidemiological survey participants [11]. The corroboration of these present study findings by other investigators supports the validity of the smoking history classification method used herein.

Multiple quantitative relationships in the current study demonstrated that patients with a positive cigarette smoking history had substantially higher arterial CoHb levels than those without a history. The mean CoHb value was markedly increased in patients with a positive cigarette smoking history. Neumann, Boehm, Cohen, Klesges, and Afsin have shown that mean or median CoHb levels are higher in cigarette smokers than in non-smokers [5-9]. The proportions of CoHb levels >1.5% and ≥2.0% in the present study were substantially greater in patients with a positive cigarette smoking history than in non-smokers. For patients in the current study with a positive cigarette smoking history, the proportion of patients with a CoHb level ≥2.0% is virtually identical to that found in smokers in another investigation [7].

The receiver operating characteristic curve is a plot depicting the true-positive and false-positive cigarette smoking history relationships among specified CoHb values. The large area under the receiver operating characteristic curve indicates that CoHb values are highly discriminated against distinguishing patients with a positive cigarette smoking history from those without a smoking history. The large area under the receiver operating characteristic curve depicting the relationship between arterial CoHb levels and cigarette smoking history herein is similar to that found in two other cohorts of trauma patients using venous CoHb values conducted by Neumann et al. [9]. Because this study used venous CoHb and investigated trauma patients with minor injuries, the major question was the applicability of the study to trauma activation patients where arterial CoHb was used [9,10]. The Neumann et al. and present study relationships between CoHb levels and cigarette smoking history are complementary.

The statistical software interpretation of the receiver operating characteristic curve indicated, through a highlighted display, that the best threshold values were CoHb >1.5% to predict a positive cigarette smoking history and CoHb ≤1.5% to predict a non-smoking history. That is, the dichotomous classification cut-off points where the sensitivity and specificity balance was found to be optimal. Using these cut-off points, sensitivity, specificity, accuracy, positive predictive, and negative predictive values exceeded 90%. The high current study accuracy indicates that a CoHb level >1.5% to identify patients with a cigarette smoking history and a CoHb level ≤1.5% to classify patients without a smoking history are reliable cut-off points (thresholds). Discriminate CoHb values for detecting cigarette smokers have been cited as 1.6-2.0% in peer-reviewed publications in two trauma cohorts and one non-trauma group [6,9].

Several factors can influence the CoHb level that is associated with daily cigarette smoking. CoHb levels have been found to be higher as the number of cigarettes smoked per day or the smoked cigarette increases; heaviness of smoking increases [5,6,9]. However, the increases in CoHb are only relatively modest for smoking >20 cigarettes per day when compared to smoking 10-20 cigarettes per day [5,6]. CoHb levels have been shown to decrease as the number of hours of abstinence since the last smoked cigarette increases; however, the CoHb level is substantially increased even with ≥12 hours of abstinence [5]. Together, these observations suggest that chronic daily cigarette smoking has a two-compartment pharmacokinetic carbon monoxide distribution effect [15]. That is, daily carbon monoxide inhalation has both a slower peripheral, extra-vascular tissue compartment distribution, e.g., myoglobin, cytochrome P-450, and cytochrome c oxidase, and a more rapid, central intravascular blood compartment distribution, e.g., CoHb [15,16]. These pharmacokinetic distribution effects likely account for exacerbated increases in CoHb levels with acute-on-chronic cigarette smoking and sustained increases in CoHb levels with short-term cigarette smoking abstinence in daily smokers.

A positive smoking history in trauma center patients has been shown to be associated with a positive blood alcohol content result, a history of alcohol misuse, and a positive toxicology result [3,4,17]. In non-trauma patient populations, there is also literature evidence that positive smoking history is associated with drug abuse disorders, decreased psychological well-being, and numerous adverse physical health effects [11,18-26]. The present study also indicates that a positive cigarette smoking history is associated with a drug abuse history in trauma activation patients aged 18-60. Of interest, data in the trauma study investigating venous CoHb levels in smokers and non-smokers demonstrated that illicit drug use in the previous 12 months was more frequent in smokers than in non-smokers [9].

As previously stated, the current study and several other investigations demonstrate that an increased CoHb level has a strong association with a positive cigarette smoking history. A rationale for obtaining an arterial blood gas sample, which includes a CoHb value, upon trauma center arrival in trauma activation patients, has been previously provided [3]. Fortunately, interventions in trauma patients have been shown to be effective in reducing chronic smoking and alcohol misuse [27,28]. For the cited smoking intervention, physician counselors educated all participants regarding the detrimental effects of smoking, the benefits of smoking cessation, and common reasons people refuse to stop smoking and offered all participants an option to use a nicotine patch [27]. Admitted patients also had a pulmonary rehabilitation nurse consultation for further counseling. For the cited alcohol misuse intervention, a clinical psychologist, in a 30-minute session, presented the participant with a comparison of their drinking habits with national norms, the participant’s blood alcohol content level at trauma center arrival, the negative social and physical consequences of alcohol misuse, the repeat trauma rates with alcohol misuse, the need for personal responsibility to manage alcohol misuse, and the treatment resources and self-help groups available in the community [28]. A letter summarizing the session was mailed to the participant following discharge.

Limitations of the study

The main limitation of the current study is that it was a retrospective analysis. Another limitation of this study is that non-cigarette smoke products were not investigated as described in an earlier publication [4].

## Conclusions

We have shown in our past investigation that a positive cigarette smoking history is associated with an alcohol misuse history in trauma activation patients aged 18-60. We have provided literature evidence in non-trauma patients that cigarette smoking is associated with chronic physical illnesses and adverse psychological well-being. The current investigation demonstrated that in trauma activation patients aged 18-60, cigarette smoking history is associated with a drug abuse history and an elevated CoHb level. We recommend that arterial blood gas be routinely assessed in trauma activation (mid- or high-level injury acuity) patients on trauma center arrival to enhance assessment of their clinical condition. If the CoHb level is elevated, the patient should undergo an assessment for alcohol misuse, drug abuse, and smoking history. A patient well-being intervention should be made available for those with any positive history.
